# Association of functional limitations and disability with elder abuse in India: a cross-sectional study

**DOI:** 10.1186/s12877-020-01619-3

**Published:** 2020-06-23

**Authors:** T. Sathya, Ramaswamy Premkumar

**Affiliations:** 1 Department of Development Studies, International Institute for Population Sciences, Govandi Station Road, Mumbai, 400088 India; 2 Arora Health Initiative, Bangalore, India

**Keywords:** Elder abuse, Disability, Functional ability, India

## Abstract

**Background:**

Globally, elder abuse is a common form of violence against the elderly. This study examines the association of disability and functional ability measures with elder abuse in India.

**Methods:**

Cross-sectional data from the UNFPA’s ‘Building Knowledge Base on Population Ageing in India’ (BKPAI 2011) have been analysed. Bivariate and multivariate logistic regression analyses have been used to examine the association of measures of disability and functional ability with elder abuse/mistreatment.

**Results:**

The overall prevalence of elder abuse in the study population is 11.4%. The prevalence of elder abuse experienced by study participants in the month before the survey is 6%. The prevalence of disability/functional ability increases the likelihood of elder abuse. Furthermore, the association between functional ability and abuse is stronger and consistent among the elderly who experienced abuse in the month preceding the survey. In addition, the association between disability and elder abuse is stronger in urban areas. Another observation is that gender introduces considerable disparities in the association of disability and functional ability measures with elder abuse. While elderly men with more than two disabilities are 1.85 times (95% CI: 1.23, 2.77, *p* < 0.003) more likely to experience abuse/mistreatment, women are 3.16 times (CI: 2.22, 4.49, *p* < .001) more exposed to it.

**Conclusions:**

The results of this study suggest a significant association of disability and functional ability with elder abuse. The association differs considerably by place of residence and gender. Measures to improve the functional health of the elderly population and measures to protect the elderly with disability and functional limitations are important in preventing abuse/mistreatment in old age.

## Background

The World Health Organization (2008) defines elder abuse as ‘a single, or repeated act, or lack of appropriate action, occurring within any relationship where there is an expectation of trust which causes harm or distress to an older person [1].’ Elder abuse varies in form and severity. It includes physical, sexual and verbal ill treatment as well as financial exploitation and neglect [2]. Elder abuse is sometimes called elder mistreatment or elder maltreatment [3].

Elder abuse has significantly adverse consequences for the health and well-being of the older population. Elder abuse leads to psychological distress and depression [4–6], mortality [7–9] and hospitalization [10]. The association between elder abuse and mortality is mainly driven by depression and lower levels of social engagement [8]. Furthermore, abuse or mistreatment in old age reduces quality of life [11, 12] as well as subjective well-being [13].

Globally, elder abuse is a common form of violence against the elderly which has significant implications on public policy. A meta-analysis consisting of data from 28 countries has reported 15.7% prevalence of elder abuse [14]. Several studies have identified major correlates of elder abuse, which include age, living arrangement, socioeconomic status and health [15–19]. Health emerges as an important and modifiable risk factor of elder abuse. Frailty is also closely linked with it [20, 21]. Mental health conditions such as depression, dementia and cognitive impairment are also significantly associated with elder abuse [22–27]. Poor functional capacity increases the risk of dependency thereby the risk of elder abuse increases. Previous tudies mainly from high-income countries have observed a significant relationship between functional ability/disability and elder abuse [19, 28–33]. A systematic review has identified functional dependence or disability as a strong risk factor of elder abuse [26]. Additionally, a meta-analysis has highlighted the differences in the prevalence of elder abuse among general population and elder with disabilities, which suggests higher prevalence of abuse among elderly with disabilities [14]. They have found that elderly persons with disability or functional limitations are at high risk of being victims of abuse because of their dependence on caregivers [30, 33–36]. Some of the risk factors related to elder abuse are cognitive and functional impairments [16], which place additional demands on caregivers [37]. Studies have shown the prevalence of abuse if caregivers are required to devote more time, particularly to elders suffering from dementia [38]. Dementia significantly increases elder abuse by caregivers due to the spending of more time with elder persons [22, 39].

In old age functional independence is an important characteristic of healthy ageing [40]. Functional limitations and disability conditions are linked to higher utilization of healthcare, lower levels of social cohesion and poor mental health status. As a result of functional limitations, the elderly are more likely to depend on others for fulfilling their basic needs Person needs assistance or adaptive equipment when he/she is faces limitations in physical capacity [30]. The functional disability is the inability or difficulty in performing everyday tasks of human beings, which are usually necessary for an independent life in social environment [41]. Moreover, elderly with functional disability greater need of help in self-care activities and more complex everyday activities and this is directly associated with elder abuse due to dependency [30]. Overall, very few studies have examined the association of health conditions such as depression, poor self-rated health and chronic diseases with elder abuse in India [15, 42–44]. However, the association of disability and functional limitations with elder abuse is less known in India.

Understanding the association of functional ability/disability with elder abuse will be useful in the context of population ageing in India. In 2011, the share of the 60+ population in India was 8.2%, which is estimated to increase to 19.4% by 2050 [45]. Increasing age is closely associated with higher risk of disabilities and low functional abilities. In this context, it is necessary to inform the policymakers about the role of functional ability and disability in determining elder abuse. In this study, we examine the association between functional ability/disability with elder abuse in India using a nationally representative data of elderly age 60 and above. We also examine to what extent the association between disability and elder abuse differs by gender and place of residence (rural/urban).

## Methods

### Data

United Nations Population Fund (UNFPA)‘s survey namely Building a Knowledge Base on Population Ageing in India (BKPAI), 2011 dataset has been used to fulfill the objectives of this study. BKPAI was an initiative to fill the knowledge gap on population ageing in India. The information gathered in this survey includes socioeconomic status; work participation and benefits; income and asset holding; living arrangement patterns and familial relations; social activities; abuse experience and nature of abuse; health status; utilisation and financing of healthcare; and reach and awareness of social security schemes among the elderly.

BKPAI was a nationally representative survey which collected data from sevenmajor demographically advanced states of India such as Himachal Pradesh, Kerala, Maharashtra, Odisha, Punjab, Tamil Nadu and West Bengal that have a higher percentage of the population in the age group 60 years and above compared to the national average. While all survey states are demographically advanced than the national average, within this sample there are significant variations in per-capita income and demographic development. The states of Punjab and Maharashtra are economically advanced states, whereas Orissa is one of the poorest states in the country. Kerala has the highest share of elderly population but also developed socio-demographic indicators [18]. The sample for each state was fixed at 1280 elderly households. From these selected households, those households that had at least one elderly member aged 60 years or above from the set of sample household and all the elderly members in the selected households were interviewed. A total of 8329 household interviews and 9852 elderly interviews were conducted in both rural and urban areas. The sample size was equally divided between urban and rural areas, irrespective of the proportion of urban and rural population. Eighty Primary Sampling Units (villages or urban wards) – 40 urban and an equal number of rural – with 16 households per Primary Sampling Unit (PSU) having an elderly person were covered in the survey. The urban and rural samples within each state were drawn separately. The PSUs in the rural areas were villages, whereas the urban wards were the PSUs in the urban areas [46].

### Outcome variable

#### Elder abuse (ever)

In the BKPAI survey, respondents were asked a question regarding their experience of abuse since they turned 60 years and in the previous month. The question was, ‘In the time since you completed 60 years of age have you faced any type of abuse or violence or neglect or disrespect by any person? ‘The respondents answered ‘yes’ or ‘no’.

#### Current experience of abuse (last 1 month)

Those who reported ever experiencing abuse were asked an additional question about the experience of abuse in the previous month. The question was, ‘Have you faced any physical or emotional abuse or violence in the last 1 month?’ A dichotomous variable was generated by taking those who said to the above question as ‘yes’ and those who never experienced abuse as ‘no’.

### Main predictors

#### ADL and IADL limitations

Data on six ADLs (activities of daily living) limitations was collected: bathing, dressing, toilet, mobility, continence and feeding. A single variable of ADL was generated by combining these six ADL and was recoded as ‘no ADL’, ‘1 ADL’ and ‘2+ ADLs. Similarly, a single variable of IADL (instrumental activities of daily living) limitations was generated by combining eight variables that included difficulty in using the telephone, shopping, preparing food, housekeeping, doing laundry, arranging transportation, handling medication and finances. These were recoded as ‘no IADL’, ‘1 IADL’ and ‘2+ IADLs’.

#### Disability

In the BKPAI survey, information on self-reported prevalence of disability was collected with the following question: ‘Do you have any of the following difficulties?’ Data on six types of disabilities was collected, namely vision, hearing, walking, teeth (chewing), speaking and memory. A single variable was generated by combining all the disability measures and recoded as ‘no disability’, ‘1 disability’ and ‘2+ disabilities’.

#### Socio-demographic characteristics

Age group (60–69, 70–79 and 80+), marital status (currently married and widowed/separated/divorced), place of residence (rural/urban), caste (Scheduled Caste or Scheduled Tribe (SC/ST), Other Backward Caste’ (OBC) and Others), religion (Hindu, Muslim, Sikh, and Others), years of schooling (0–4 years, 5–9 years and 10+ years), wealth quintile (poorest, poorer, middle, richer and richest).

### Statistical analysis

Bivariate analysis has been carried out to understand the sample distribution and the prevalence of elder abuse by disability and functional limitations. Logistic regression has been used to examine the association of socio-demography and disability/functional limitations with elder abuse. The regression analysis has been stratified by gender and place of residence to better understand the difference in association across male, female, rural and urban. All statistical analyses have been performed using STATA 12 (StataCorp, LP, College Station, Texas).

## Results

Table 1 describes the characteristics of the study population. The overall prevalence of elder abuse in the study population is 11.4%. The prevalence of elder abuse experienced by study participants in the month before the survey is 6%. In this study, the percentage of older adults with no disability is 82.1, while those with at least one disability is 12.6%. The share of women participants (52.6) is higher in the total sample and a higher share of study participants is from rural areas (73.5%). About 60% of the study population is currently married. While 3% of study participants have reported difficulties in any one ADL, 4.79% have reported difficulties in two or more than two ADLs. The percentage of elderly with 1+ IADL is 72.5. About two-thirds of study participants have had only 0–4 years of schooling.

**Table 1:** Characteristics of the study population, BKPAI, 2011

Characteristics	Categories	Percent
Abused since 60 years old	No	88.6
	Yes	11.4
Abused in Last one Month	No	94.79
	Yes	6.02
ADL	No	92.37
	1 ADL	2.83
	2 + ADL	4.79
IADL	No IADL	12.03
	1 IADL	15.45
	2 + IADL	72.52
Disability	No disability	82.13
	1 disability	12.58
	2 + disability	5.29
Age	60–69	61.85
	70–79	27.3
	80+	10.85
Sex	Male	47.33
	Female	52.67
Marital status	Married	60.34
	Others	39.66
Residence	Rural	73.57
	Urban	26.43
Caste	SC/ST	26.37
	OBC	36.71
	Others	36.92
Religion	Hindu	78.25
	Muslim	8.34
	Sikh	9.15
	Others	4.26
Schooling	0–4 years	64.43
	5–9 years	20.46
	10+ years	15.1
Wealth quintile	Poorest	24.25
	Second	22.14
	Middle	20.46
	Fourth	18.36
	Richest	14.79
State	Himachal Pradesh	15.03
	Punjab	13.92
	West Bengal	12.94
	Orissa	15.03
	Maharashtra	14.57
	Kerala	13.86
	Tamil Nadu	14.66

**Fig. 1:**
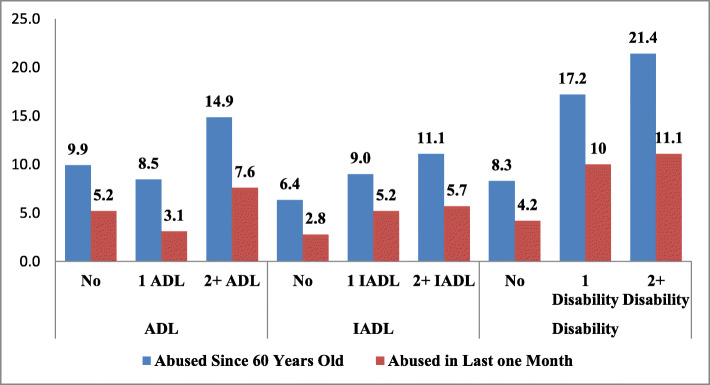
Prevalence of elder abuse by ADL, IADL and disabilities, BKPAI

Figure 1 shows the prevalence of elder abuse by ADL and IADL limitations and disability conditions. The prevalence of elder abuse is 9.4% among the elderly with no ADL limitations and increases to 14.9% with 2+ ADL limitations. The prevalence of abuse in last 1 month increases with ADL, IADL and disability conditions. Similarly, the elderly with no IADL limitations have lower prevalence of elder abuse of 6.4%, which increases to 11% for the elderly with 2+ IADLs. The prevalence of elder abuse with disability increases from 17.2% for elderly with one disability to 21.4% with 2+ disabilities. Similarly, the prevalence of abuse in last 1 month increases with ADL, IADL and disability conditions.

Table 2 shows the logistic regression results of elder abuse in relation to disability and functional limitations along with socio-demographic characteristics. The association between disabilities and elder abuse is positive and significant. The elderly with 2+ disabilities are 2.47 times more likely to experience abuse [CI = 1.90, 3.21, *p* < .001]. Similarly, the elderly with 2+ ADLs are 40% more likely to experience abuse. The association of disability and functional ability with abuse in the month preceding the survey was consistent and significant. The elderly with 2+ IADLs and disability were two times more likely to experience abuse. Similarly, the elderly with 2+ ADLs had an 83% higher risk of experiencing abuse in the preceding month. The association between marital status and abuse significant only those who ever experienced (Abused since 60 years old). Currently married elders have lower risks of abuse than others [OR = 1.24, CI = 1.05, 1.48, *p* < .001]. Socioeconomic status is negatively associated with elder abuse. The elderly individual with 10+ years of schooling is less likely to experience abuse in last 1 month as well as ever experience of abuse (since 60 years old). The association between wealth quintile and elder abuse is highly significant across measures of abuse. The elderly in the richest wealth quintile are 70% less likely to experience abuse (since 60 years old) [CI = 0.22, 0.40, *p* < .001] and abuse in last 1 month [CI = 0.18, 0.48, *p* < .001]. The elderly in Maharashtra are more likely to experience abuse than those in Himachal Pradesh. On the other hand, the elderly in West Bengal, Odisha, Kerala and Tamil Nadu are less likely to experience abuse as compared to those in Himachal Pradesh. In particular, the prevalence of abuse in the month preceding the survey was 23 times higher in Maharashtra.

**Table 2:** Logistic regression results of disability and functional health with elder abuse, BKPAI, 2011

Characteristics	Abused since 60 years old	Abused in last one month
	OR (95% CI)	OR (95% CI)
**ADL**		
No ADL	Ref	Ref
1 ADL	1.00[0.61, 1.64]	0.66 [0.26, 1.65]
2 + ADL	1.31*[0.96, 1.80]	1.83**[1.12, 2.99]
**IADL**		
No IADL	Ref	Ref
1 IADL	1.28 [0.94, 1.74]	1.92***[1.21, 3.06]
2 + IADL	1.40**[1.07, 1.83]	2.20***[1.46, 3.31]
**Disability**		
No disability	Ref	Ref
1 disability	1.93***[1.59, 2.34]	2.34***[1.75, 3.13]
2 + disability	2.47***[1.90, 3.21]	2.35***[1.56, 3.53]
**Age**		
60–69	Ref	Ref
70–79	0.9 [0.75, 1.07]	0.77*[0.59, 1.01]
80+	1.14[0.89, 1.45]	0.97[0.66, 1.43]
**Sex**		
Male	Ref	Ref
Female	0.93[0.78, 1.10]	0.99[0.76, 1.29]
**Marital status**		
Married	Ref	Ref
Others	1.24**[1.05, 1.48]	1.19[0.91, 1.55]
Residence		
Rural	Ref	Ref
Urban	0.94[0.79, 1.11]	1.08[0.83, 1.39]
**Caste**		
SC/ST	Ref	Ref
OBC	1.01[0.81, 1.25]	0.80 [0.58, 1.11]
Others	1.37***[1.13, 1.66]	1.47*** [1.10, 1.97]
**Religion**		
Hindu	Ref	Ref
Muslim	0.95[0.69, 1.30]	0.95[0.63, 1.43]
Sikh	1.42*[0.98, 2.03]	0.67[0.15, 2.94]
Others	1.08[0.74, 1.58]	0.98[0.61, 1.57]
**Schooling**		
0–4 years	Ref	Ref
5–9 years	0.89[0.72, 1.10]	0.78[0.57, 1.07]
10+ years	0.64***[0.48, 0.86]	0.59**[0.37, 0.94]
**Wealth quintile**		
Poorest	Ref	Ref
Second	0.64***[0.52, 0.80]	0.59***[0.44, 0.79]
Middle	0.43***[0.34, 0.56]	0.39***[0.27, 0.56]
Fourth	0.35***[0.27, 0.46]	0.34***[0.23, 0.50]
Richest	0.30***[0.22, 0.40]	0.29***[0.18, 0.48]
**State**		
Himachal Pradesh	Ref	Ref
Punjab	0.87[0.62, 1.24]	0.28*[0.07, 1.03]
West Bengal	0.52***[0.38, 0.71]	1.48[0.82, 2.68]
Orissa	0.55***[0.41, 0.74]	0.83[0 .43, 1.59]
Maharashtra	3.71***[2.93, 4.69]	23.45***[14.32, 38.40]
Kerala	0.41***[0.28, 0.61]	1.01[0.47, 2.15]
Tamil Nadu	0.15***[0.09, 0.24]	0.43* [0.18, 1.02]

*OR* Odds Ratio, *Ref* reference; ***Significant at *p* < .001, **Significant at *p* < .005, *Significant at *p* < .01

Table 3 shows the logistic regression results of elder abuse stratified by gender. The association with elder abuse of disability, functional limitations and years of schooling differs considerably by gender**.** The association of disability with elder abuse is significantly stronger for women than for men. Elderly women with one disability are 2.18 times more likely to experience abuse [CI = 1.68, 2.84, *p* < .001] and those with 2+ disabilities are 3.16 times more likely to experience abuse (abused since 60 years old) [CI = 2.22, 4.49, *p* < .001]. The association between IADL and abuse (abused since 60 years old) significant only for women. Similarly, elderly women with 2+ disabilities were three times more likely to experience abuse in the month before the survey [*p* < .001]. Elderly men and women with IADL limitations had higher risk of abuse in this period. However, the association between ADL limitations and abuse in last 1 month is significant only for men. The positive association between age (80+) and abuse (abused since 60 years old) is significant only for men.

**Table 3:** Logistic regression results of elder abuse stratified by gender differences, BKPAI, 2011

Characteristics	Abused since 60 years old		Abused in last one month	
	Male	Female	Male	Female
	OR (95% CI)	OR (95% CI)	OR (95% CI)	OR (95% CI)
**ADL**				
No ADL	Ref	Ref	Ref	Ref
1 ADL	0.89[0.41, 1.90]	1.06[0.55, 2.05]	0.50[0.10, 2.32]	0.81[0.25, 2.61]
2 + ADL	1.60*[0.99, 2.59]	1.14[0.75, 1.74]	2.81***[1.37,5.78]	1.41[0.71, 2.81]
**IADL**				
No IADL	Ref	Ref	Ref	Ref
1 IADL	1.07[0.67, 1.71]	1.40**[0.92, 2.13]	1.92*[0.96, 3.81]	1.83*[0.96,3.49]
2 + IADL	1.22[0.81, 1.83]	1.47**[1.03, 2.10]	1.69*[0.92, 3.13]	2.56***[1.46,4.49]
**Disability**				
No disability	Ref	Ref	Ref	Ref
1 disability	1.70***[1.27,2.28]	2.18***[1.68,2.84]	2.12***[1.36,3.29]	2.63***[1.77, 3.90]
2 + disability	1.85***[1.23,2.77]	3.16***[2.22,4.49]	1.72*[0.92, 3.22]	3.18***[1.83, 5.50
**Age**				
60–69	Ref	Ref	Ref	Ref
70–79	0.95[0.74, 1.23]	0.84[0.66, 1.07]	0.76[0.51, 1.14]	0.72*[0.49, 1.05]
80+	1.56**[1.10, 2.21]	0.86[0.61, 1.21]	1.07[0.59, 1.92]	0.85[0.50, 1.42]
**Marital status**				
Married	Ref	Ref	Ref	Ref
Others	1.04[0.79, 1.39]	1.34***[1.07,1.68]	0.89[0.56, 1.42]	1.34*[0.96, 1.88]
**Caste**				
SC/ST	Ref	Ref	Ref	Ref
OBC	1.17[0.85, 1.60]	0.88[0.65, 1.18]	1.15[0.71, 1.87]	0.56**[0.36, 0.88]
Others	1.38**[1.03, 1.84]	1.39**[1.07, 1.80]	1.77**[1.12, 2.78]	1.32[0.89, 1.95]
**Religion**				
Hindu	Ref	Ref	Ref	Ref
Muslim	1.01[0.63, 1.62]	0.89[0.58, 1.36]	1.11[0.62, 2.00]	0.84[0.47, 1.50]
Sikh	1.74**[1.01, 2.97]	1.23[0.75, 2.00]	0.37[0.03, 3.99]	1.06[0.15, 7.16]
Others	0.67[0.34, 1.31]	1.43[0.89, 2.29]	0.60[0.25, 1.42]	1.20[0.66, 2.17]
**Schooling**				
0–4 years	Ref	Ref	Ref	Ref
5–9 years	1.01[0.77, 1.32]	0.76[0.54, 1.06]	0.77[0.50, 1.17]	0.83[0.51, 1.35]
10+ years	0.72*[0.51, 1.03]	0.43***[0.23,0.77]	0.63[0.36, 1.11]	0.39*[0.13, 1.18]
**Wealth quintile**				
Poorest	Ref	Ref	Ref	Ref
Second	0.58***[0.42,0.81]	0.67***[0.50,0.89]	0.62**[0.39, .96]	0.56***[0.38, 0.84]
Middle	0.41***[0.29,0.59]	0.42***[0.31,0.59]	0.37***[0.22, .63]	0.44***[0.28, 0.69]
Fourth	0.35***[0.24,0.51]	0.33***[0.23,0.46]	0.34***[0.19, .61]	0.33***[0.20, 0.53]
Richest	0.30***[0.19,0.46]	0.28***[0.19,0.42]	0.29***[0.14, 0.59]	0.31***[0.16, 0.58]
**State**				
Himachal Pradesh	Ref	Ref	Ref	Ref
Punjab	0.81[0.48, 1.36]	0.92[0.58, 1.48]	0.52[0.08, 3.09]	0.17*[0.02, 1.09]
West Bengal	0.59**[0.38, 0.92]	0.46***[0.30,0.71]	1.90[0.78, 4.63]	1.21[0.55, 2.66]
Orissa	0.53***[0.34,0.81]	0.55***[0.36,0.84]	1.15[0.45, 2.93]	0.61[0.24, 1.51]
Maharashtra	3.53***[2.52,4.94]	3.78***[2.72,5.24]	24.11***[11.3,51.24]	23.23***[12.0,44.6]
Kerala	0.29***[0.15,0.56]	0.53**[0.32, 0.87]	0.93[0.27, 3.24]	1.14[0.43, 3.01]
Tamil Nadu	0.13***[0.06,0.27]	0.16***[0.89,0.30]	0.25*[0.05, 1.24]	0.57[0.19, 1.64]

*OR* Odds Ratio, *Ref* reference; ***Significant at *p* < .001, **Significant at *p* < .005, *Significant at *p <* .01

The association between marital status and elder abuse is significant for women. Elderly women with 10+ years of education have less likelihood of experiencing abuse. The association between wealth quintile and elder abuse is highly significant and negative for both men and women. Elderly men and women in the richest wealth quintile are less likely (up to 70%) to experience abuse across two measures (abused since 60 years old and in last 1 month). Table 4 shows the logistic regression results of ever experiencing abuse and abuse experienced in the month before the survey by place of residence. The association between disability and abuse differs considerably for urban and rural areas. A strong evidence of higher risk of abuse is observed among the elderly in urban areas with disability. The elderly in urban areas with 2+ disabilities are three times more likely to experience abuse since 60 years old [OR = 3.49, CI = 2.38, 5.13, *p* < .001]. Whereas, elderly residing in rural areas with 2+ disabilities are 1.85 times more likely to experience elder abuse since 60 years old [CI = 1.28,2.67, *p* < .001]. The elderly in rural areas with 2+ ADL limitations had higher risk of abuse in the month preceding the survey. The association between IADL and abuse (in last 1 month) is significant in rural and urban areas. Religion and marital status is significant only in rural areas. The association between education and elder abuse is significant only in urban areas. The elderly in the richest wealth quintile category have less likelihood of experiencing abuse in both rural and urban areas.

**Table 4:** Logistic regression results of elder abuse stratified by place of residence, BKPAI, 2011

	Abused since 60 years old		Abuse in last one month	
	Rural	Urban	Rural	Urban
Characteristics	OR (95% CI)	OR (95% CI)	OR (95% CI)	OR (95% CI)
**ADL**				
No ADL	Ref	Ref	Ref	Ref
1 ADL	0.60[0.28, 1.30]	1.06[0.81, 3.13]	0.37[0.08, 1.72]	1.08[0.32, 3.67]
2 + ADL	1.46*[0.98, 2.16]	1.13[0.67, 1.91]	2.46***[1.34, 4.51]	1.03[0.42, 2.51]
**IADL**				
No IADL	Ref	Ref	Ref	Ref
1 IADL	1.08[0.70, 1.67]	1.47*[0.94, 2.28]	1.56[0.80, 3.04]	2.24**[1.15, 4.34]
2 + IADL	1.38*[0.95, 1.99]	1.29[0.87, 1.92]	2.14***[1.21, 3.77]	2.17**[1.18, 3.99]
**Disability**				
No disability	Ref	Ref	Ref	Ref
1 disability	1.56***[1.21, 2.01]	2.63***[1.94, 3.57]	2.00***[1.36, 2.94]	2.90***[1.84, 4.57]
2 + disability	1.85***[1.28, 2.67]	3.49***[2.38, 5.13]	1.91**[1.08, 3.37]	3.07***[1.67, 5.65]
**Age**				
60–69	Ref	Ref		
70–79	0.80*[0.64, 1.00]	1.12[0.84, 1.49]	0.64**[0.45, 0.92]	1.00[0.65, 1.54]
80+	1.08[0.79, 1.47]	1.28[0.86, 1.90]	0.99[0.60, 1.63]	0.99[0.52, 1.86]
**Sex**				
Male	Ref	Ref	Ref	Ref
Female	0.92[0.74, 1.14]	0.96[0.72, 1.27]	1.00[0.71, 1.41]	0.99[0.65, 1.53]
**Marital status**				
Married	Ref	Ref	Ref	Ref
Others	1.34***[1.08, 1.66]	1.11[0.83, 1.47]	1.30[0.92, 1.82]	1.07[0.70, 1.63]
**Caste**				
SC/ST	Ref	Ref	Ref	Ref
OBC	0.99[0.75, 1.29]	1.08[0.75, 1.55]	0.74[0.49, 1.12]	0.94[0.55, 1.60]
Others	1.36**[1.07, 1.73]	1.46**[1.05, 2.02]	1.51**[1.04, 2.20]	1.60*[0.98, 2.61]
**Religion**				
Hindu	Ref	Ref	Ref	Ref
Muslim	0.92[0.58, 1.48]	0.95[0.61, 1.48]	0.76[0.38, 1.52]	1.08[0.63, 1.85]
Sikh	2.13***[1.22, 3.73]	1.11[0.65, 1.91]	0.32[0.02, 4.20]	1.17[0.19, 7.24]
Others	1.28[0.75, 2.17]	0.95[0.54, 1.66]	1.23[0.64, 2.35]	0.83[0.39, 1.74]
**Schooling**				
0–4 years	Ref	Ref	Ref	Ref
5–9 years	1.02[0.77, 1.35]	0.75**[0.55, 1.03]	0.89[0.57, 1.38]	0.68[0.43, 1.07]
10+ years	0.75[0.48, 1.17]	0.55***[0.37, 0.82]	0.50[0.21, 1.18]	0.59*[0.32, 1.06]
**Wealth quintile**				
Poorest	Ref	Ref	Ref	Ref
Second	0.63***[0.49, 0.81]	0.69[0.44, 1.09]	0.60***[0.42, .85]	0.64[0.34, 1.17]
Middle	0.40***[0.29, 0.56]	0.52***[0.34, 0.81]	0.47***[0.29, 0.77]	0.37***[0.20, 0.68]
Fourth	0.34***[0.23, 0.48]	0.40***[0.26, 0.63]	0.35***[0.19, 0.63]	0.36***[0.19, 0.67]
Richest	0.30***[0.19, 0.47]	0.33***[0.20, 0.53]	0.31**[0.12, 0.79]	0.30***[0.15, 0.59]
**State**				
Himachal Pradesh	Ref	Ref	Ref	Ref
Punjab	0.53**[0.30, 0.93]	1.29[0.79, 2.09]	0.40[0.06, 2.65]	0.25[0.04, 1.46]
West Bengal	0.48***[0.32, 0.72]	0.64*[0.38, 1.05]	1.47[0.67, 3.23]	1.74[0.69, 4.37]
Orissa	0.53***[0.36, 0.77]	0.60*[0.36, 1.00]	1.22[0.55, 2.71]	0.38[0.10, 1.35]
Maharashtra	3.23***[2.40, 4.36]	4.85***[3.25, 7.24]	24.5***[13.0,46.4]	24.75***[11.0, 55.2]
Kerala	0.31***[0.18, 0.53]	0.61[0.33, 1.10]	0.92[0.32, 2.57]	1.19[0.37, 3.77]
Tamil Nadu	0.17***[0.10, 0.30]	0.10***[0.39, 0.28]	0.37[0.11, 1.22]	0.58[0.15, 2.15]

*OR* Odds Ratio, *Ref* reference; ***Significant at *p* < .001, **Significant at *p* < .005, *Significant at *p* < .01

## Discussion

This study examines the relationship of disability and functional limitation with mistreatment/abuse among the elderly using a nationally representative data of the elderly population in India. It also assesses the differences in the association by gender and place of residence. Findings reveal that the prevalence of abuse increases with functional limitations and disability. The regression results suggest a significant relationship between disability/functional limitations and elder abuse. In particular, the association between disability and elder abuse is stronger than ADL and IADL limitations. Furthermore, the results differ considerably for the conditions of ever having experienced abuse and having experienced abuse in the month preceding the survey. The association is slightly stronger and consistent across functional ability measures (ADL and IADL) for the elderly who experienced abuse in the month before the survey. Additionally, the association is stronger for women and urban residents.

In this study, the association of disability/functional limitations and sociodemographic variables with elder abuse differ by ever experience of abuse and having experienced abuse in the month preceding the survey. Factors such as disability/functional limitations, caste and schooling is associated with elder abuse across two time period. Marital status and caste significant only among those who experienced abuse since 60 years old. The association of schooling and poor self-rated health with elder abuse/mistreatment is significant only in urban areas. On the other hand, marital status, caste, religion, tobacco use were associated with elder abuse/mistreatment in rural areas.

Previous literature has indicated that health factors such as disabilities, dementia and chronic diseases are associated with elder abuse. More specifically, conditions like dementia and frailty are closely associated with elder abuse suggesting the role of situationnel approach theory that hypothesizes the overburden on caregivers resulting in higher degrees of abuse [16, 22]. In this study, the prevalence of disability conditions and functional limitations are positively associated with elder abuse/ill-treatment as consistent with literature [19, 28–33]. Previous literature has shown that disability in old age is associated with long-term care and care dependence [47] as well higher healthcare expenditure [48]. This in turn may lead to caregivers and family members abusing their elders. Elders with disabilities and functional limitations find it difficult to carry out day-to-day activities efficiently, which generates stress in the household and demands care from other family members, thereby increasing the risk of abuse or mistreatment. Studies have observed higher degree of abuse by care providers of elders suffering from dementia mainly as a result of caregiver stress [22, 36, 49–51].

It is also important to observe that the elderly, especially women and those who residing in urban areas are at higher risk of elder abuse than their rural counterparts. Previous studies have shown that women with disability are at higher risk of elder abuse than men [52–54]. This indicates the gender vulnerability in reference to elder abuse in India. Previous studies have suggested that women experience greater health problems such as disability, functional limitations and psychological distress than men [55, 56]. In addition, widowhood is one of the important contributors to poor health outcomes [57] as a larger share of women are widows and experience longer durations of widowhood in old age. As a result of this, women in old age are more likely to depend on others for financial support and experience poor mental health status. Due to a wider age gap between husband and wife, most women experience old age as widows. Previous studies conducted in India have shown that widowed women are at high risk of experiencing violence and abuse [58]. The gender gap in employment in old age is also important to note. Elderly women in India are less likely to be employed [59], which increases their financial dependency and elder abuse [60]. Furthermore, the elderly in urban areas are at higher risk of elder abuse than their rural counterparts. The results are consistent with previous studies which have suggested that the urban elderly are at higher risk of abuse [61].

In this study, the association between socioeconomic status and elder abuse is significant and negative, which is consistent with previous literature [18, 52]. Studies have shown that socioeconomic status plays an important role in several ways which may prevent elder abuse [19]. A study conducted in Tamil Nadu has pointed out a significant protective impact of the wealth index on elder abuse [15]. Another study conducted in South Korea has reflected similar results [52].

The results of this study highlight the role of disability and functional limitations and elder abuse highlighting the significance of functional ability. While the prevalence of disability and ADL limitations increases with age, the negative consequences associated with disability highlight the need for healthcare to address the problem more efficiently. Further, the study identifies the subset of vulnerable population. The association differs by place of residence and gender. This study identifies women as the more vulnerable population in India than men. This may be partly due to the age difference between husband and wife leading to women living for more years as widows. Chances of men living as widowers is less likely. This may result in higher risk of elder abuse of women [18, 62].

### Strengths

To our knowledge, this is the first study to examine the role of multiple disabilities and functional limitations on elder abuse in India. The association of disability and functional limitations with abuse is consistent and strong. Most of the previous studies have used ever experience of abuse/mistreatment. In this study, we use the conditions of ever experience of abuse and current experience of abuse. It is important to note that 6 % of elderly in India experienced abuse in the month preceding the survey. In this context, a more detailed investigation of abuse is important. Furthermore, the study uses nationally representative data. Therefore, the results can be generalized at the national level.

### Limitation of the study

Elder abuse record is self-reported which may have some response bias. This reporting bias could also affect the result. It is also notable that the assessment of elder abuse is based on a single question. Furthermore, most of the previous studies have used past year prevalence of elder abuse and our study covered lifetime (since participants turned 60 years old) duration as well as abuse in last 1 month. Therefore this study has some limitation while comparing with other studies. This study included only the overall prevalence of abuse and abuse in last 1 month with yes or no options. The severity of the abuse was not captured in the survey [63]. Furthermore, the result is based on only selected states of India. In this perspective, more research is needed to understand the prevalence and correlates of elder abuse across states of India. Additionally, the result of this study is based on cross-sectional data. Therefore, any causal relationship cannot established.

## Conclusions

Elder abuse is a human rights issue and preventing it necessary to improve the overall wellbeing of the growing elderly population in India. In this study, elder abuse is linked with disability and functional limitation among the elderly in India. The association is particularly stronger among women and urban residents. It suggests the differences in the association across population subgroups. Elder abuse violates the social norms of respecting elders. Disability and multi-disability are strongly associated with elder abuse. In this context, it is necessary to improve the recognition of elder abuse as a public health concern. Also, it is important to improve research to understand the factors involved in elder abuse and to develop strategies for its prevention. Furthermore, the government’s welfare and protection measures and programmes should support elders with disability. The immediate need is to properly protect disabled elders from abuse.

## Data Availability

The data-sets used in the present study are available from the corresponding author on request.

## Abbreviations

ADL: Activities of daily living
BKPAI: Building Knowledge Base on Population Ageing in India
CI: Confidence interval
IADL: Instrumental activities of daily living
OBC: Other Backward Class
OR: Odds ratio
SC: Scheduled caste
UNFPA: United Nations Population Fund
